# Lumbar Stabilization and Gluteal Strengthening Exercises Versus Health Education for Chronic Low Back Pain: A Retrospective Observational Study

**DOI:** 10.7759/cureus.102555

**Published:** 2026-01-29

**Authors:** Akshyaa T Rekha, Karthikeyan Kulothungan, Rekha Kanagamuthu, Praveen Malaiyandi, Tamilarasan Muniyapillai, Bala Vaishnavi S, Akalya Elango

**Affiliations:** 1 Community Medicine, Dhanalakshmi Srinivasan Medical College and Hospital, Siruvachur, IND; 2 Physiology, Srinivasan Medical College and Hospital, Dhanalakshmi Srinivasan University, Samayapuram, IND; 3 Orthopedics, Srinivasan Medical College and Hospital, Dhanalakshmi Srinivasan University, Samayapuram, IND

**Keywords:** chronic pain, disability evaluation, exercise therapy, low back pain, muscle strengthening, physical therapy modalities

## Abstract

Background and aim

Chronic low back pain represents a substantial global health burden with escalating prevalence rates and significant disability implications. Contemporary clinical practice guidelines emphasize exercise-based interventions as first-line conservative management strategies. However, optimal exercise prescription protocols remain under investigation. This study aimed to compare functional disability outcomes between patients receiving combined gluteus muscle strengthening with lumbar stabilization exercises versus those receiving spine-neutral health education alone for chronic low back pain management, utilizing the Oswestry Disability Index (ODI) as the primary outcome measure.

Methodology

This retrospective, observational, comparative study was conducted at a tertiary care teaching hospital, analyzing treatment outcomes in 50 participants aged 18-45 years with chronic low back pain and ODI scores of 5 or higher who received physiotherapy management between August 2023 and February 2024. Patients were stratified into group A (n = 25), who received spine-neutral health training, or group B (n = 25), who received combined gluteus muscle strengthening and lumbar stabilization exercises plus health education, based on initial clinical assessment and therapeutic protocol assignment during routine clinical practice. Treatment protocols were implemented over four weeks with alternate-day exercise sessions for group B participants. Baseline and post-intervention assessments included demographic characterization, occupational classification, educational attainment, and ODI scoring. Statistical analysis employed independent samples t-tests, paired-samples t-tests, and chi-square tests with significance established at an alpha level of 0.05.

Results

Baseline demographic characteristics demonstrated homogeneity between groups, with mean ages of 28.48 years in group A and 27.80 years in group B without statistically significant differences. Both intervention modalities achieved significant within-group ODI reductions, with group A demonstrating a mean decrease from 12.28 to 6.60, representing a reduction of 5.68 points, and group B showing a mean decrease from 13.68 to 4.76, representing a reduction of 8.92 points, both achieving statistical significance with probability values below 0.0001. Between-group comparison revealed statistically significant superiority of the combined exercise protocol, with post-intervention ODI scores of 6.60 in group A compared to 4.76 in group B, with a probability value of 0.0433. Clinical success rates, defined as 50% or greater disability reduction, were achieved by 12 participants (48.00%) in group A compared to 20 participants (80.00%) in group B, with statistical significance. Complete functional recovery was attained by seven participants (28.00%) in group A compared to 12 participants (48.00%) in group B. Severe disability classification was completely eliminated across both intervention groups.

Conclusion

Combined gluteus muscle strengthening and lumbar stabilization exercises demonstrated superior therapeutic efficacy compared to spine-neutral health education alone, achieving significantly greater functional disability reduction and clinical success rates. Both interventions produced clinically meaningful improvements, supporting conservative management approaches as first-line treatment strategies for chronic low back pain rehabilitation.

## Introduction

Chronic low back pain represents a substantial global health burden, with prevalence rates demonstrating a persistent upward trajectory over recent decades. The Global Burden of Disease Study 2017 documented that low back pain constituted one of the leading causes of years lived with disability worldwide, affecting millions of individuals across diverse demographic and occupational groups [1]. Epidemiological projections suggest that the burden of low back pain will continue to escalate through 2050, necessitating evidence-based therapeutic interventions that address both immediate symptomatology and long-term functional restoration [2]. Contemporary clinical practice guidelines emphasize the importance of multimodal, non-pharmacological approaches in the primary care management of chronic low back pain, with exercise-based interventions forming a cornerstone of conservative treatment strategies [3,4].

Among exercise modalities, lumbar stabilization exercises have emerged as a particularly promising therapeutic approach, targeting the deep spinal muscles that provide segmental control and postural support [5]. These exercises focus on enhancing neuromuscular control of the lumbar spine through activation of the transversus abdominis, multifidus, and other core musculature, thereby improving spinal stability and reducing pain-related disability [6]. Multiple systematic reviews and meta-analyses have demonstrated the efficacy of stabilization exercises in reducing pain intensity and improving functional outcomes in patients with chronic low back pain, with effect sizes generally favoring stabilization protocols over conventional exercise programs [7,8]. The theoretical foundation for lumbar stabilization training rests upon the premise that patients with chronic low back pain exhibit impaired proprioceptive acuity and diminished neuromuscular control, which can be ameliorated through targeted exercise interventions [9].

Complementing lumbar stabilization approaches, gluteus muscle strengthening has garnered increasing attention in the rehabilitation of chronic low back pain, particularly given the biomechanical interdependence between lumbopelvic stability and hip muscular function [10]. The gluteal muscles, comprising the gluteus maximus, medius, and minimus, play critical roles in maintaining pelvic alignment, controlling hip rotation, and distributing mechanical loads across the lumbopelvic complex [11]. Weakness or dysfunction of the gluteal musculature has been implicated in aberrant movement patterns that may perpetuate or exacerbate low back pain symptoms [12]. Recent investigations have demonstrated that gluteus muscle strengthening, when integrated with core stabilization protocols, produces superior outcomes in pain reduction and functional improvement compared to stabilization exercises alone [13]. The synergistic effects of combined lumbar stabilization and gluteal strengthening appear to address multiple biomechanical deficits simultaneously, offering a comprehensive therapeutic approach for chronic low back pain management [14,15].

Despite the growing body of evidence supporting exercise-based interventions, questions remain regarding optimal exercise prescription, the comparative effectiveness of different exercise protocols, and the mechanisms underlying therapeutic benefits. The present study aimed to compare functional disability outcomes between patients who received structured protocols combining gluteus muscle strengthening with lumbar segmental stabilization exercises versus those who received spine-neutral health education alone during routine clinical physiotherapy management.

## Materials and methods

Study design and setting

This investigation employed a retrospective, observational, comparative study design to evaluate functional disability outcomes among patients with chronic low back pain who received either gluteus muscle strengthening combined with lumbar stabilization exercises or spine-neutral health education as part of routine physiotherapy clinical practice. This study was conducted through retrospective analysis of treatment records from the Department of Physiotherapy at Dhanalakshmi Srinivasan Medical College and Hospital, Perambalur, Tamil Nadu, India, examining patients who received treatment between August 2023 and February 2024.

Study period

Physiotherapy interventions were implemented between August 2023 and February 2024, with each patient completing a standardized four-week treatment protocol according to departmental clinical practice guidelines. Following completion of all treatment protocols and post-intervention assessments by March 2024, retrospective data extraction and statistical analysis were conducted between April 2024 and June 2024. Data collection systematically abstracted baseline clinical assessments, demographic characteristics, treatment protocol assignments, and post-treatment functional disability evaluations from existing clinical documentation.

Ethics committee approval

Ethical clearance for this retrospective observational study was obtained from the Institutional Ethics Committee of Dhanalakshmi Srinivasan Medical College and Hospital (#IECHS/IRCHS.No.416) prior to data extraction and analysis, ensuring compliance with the Declaration of Helsinki principles and Good Clinical Practice guidelines. The ethical approval encompassed a comprehensive review of the retrospective study protocol, waiver of informed consent for retrospective data analysis, patient confidentiality protection measures, data anonymization procedures, and secure data storage protocols. All treatment protocols analyzed were part of routine clinical care, and patients provided standard clinical consent for treatment and for the anonymized use of their data for quality improvement and research purposes at the time of initial clinical consultation, ensuring adherence to principles of patient autonomy and confidentiality throughout the retrospective data analysis process.

Inclusion criteria

Participants were eligible for study enrollment if they satisfied the following criteria: adults aged 18-45 years presenting with chronic low back pain defined as persistent or recurrent pain localized between the 12th rib and gluteal folds lasting a minimum of three months duration, Oswestry Disability Index (ODI) score of 5 or higher indicating at least mild functional disability attributable to low back pain, willingness to participate in the assigned intervention protocol for the complete study duration, and capacity to provide informed consent and complete self-reported outcome measurement instruments. These inclusion parameters ensured recruitment of a homogeneous study population with clinically significant functional impairment amenable to conservative exercise-based interventions while maintaining sufficient disability burden to detect meaningful therapeutic improvements.

Exclusion criteria

Prospective participants were excluded from study enrollment based on the following criteria: age below 18 years or above 45 years; presence of specific spinal pathology including vertebral fractures, malignancy, infection, inflammatory spondyloarthropathies, or cauda equina syndrome requiring urgent medical intervention; previous spinal surgery or planned surgical intervention during the study period; neurological deficit or radiculopathy with motor weakness; pregnancy or immediate postpartum period; severe cardiopulmonary conditions precluding exercise participation; cognitive impairment or psychiatric disorders limiting capacity for informed consent or protocol adherence; concurrent participation in other therapeutic interventions or clinical trials; and unwillingness to comply with the assigned intervention protocol or complete outcome assessments. These exclusion parameters ensured participant safety, minimized confounding variables, and optimized internal validity by restricting enrollment to patients with non-specific mechanical low back pain appropriate for conservative management.

Sample size estimation

Retrospective sample size justification was performed utilizing the formula for comparison of two means based on anticipated differences in ODI scores between treatment groups, ensuring adequate statistical power for comparative analysis. Jeong et al. reported in their study of 30 patients with chronic low back pain that combined gluteus muscle strengthening and lumbar stabilization exercises produced mean ODI score reductions of approximately 8.5 points, with a standard deviation of 4.2 points [10]. Employing these parameters as reference values and assuming a clinically meaningful between-group difference of 4.0 points in ODI score reduction, with alpha error probability of 0.05 for two-tailed testing and desired statistical power of 80% (beta error: 0.20), the sample size was calculated using the formula below. This calculation yielded approximately 22 participants per group.

$n = \frac{2(Z_\alpha + Z_\beta)^2 \sigma^2}{d^2}$

Accounting for potential attrition and non-compliance estimated at 10%, the final sample size was determined to be 25 participants per intervention group, constituting a total study population of 50 participants. This sample size provided adequate statistical power to detect clinically significant between-group differences while maintaining feasibility constraints related to recruitment capacity and resource availability.

Sampling method

Retrospective case identification was accomplished through a systematic review of the physiotherapy department's clinical records from August 2023 to February 2024. All patients meeting eligibility criteria who completed chronic low back pain treatment protocols during this period were identified through electronic medical record screening and departmental physiotherapy registers. Treatment group assignments were determined during routine clinical practice based on initial physiotherapist assessment, clinical presentation severity, patient functional capacity, and individualized treatment planning protocols established within the department. Group A patients (n = 25) received spine-neutral health education training as their primary conservative management approach, while group B patients (n = 25) received combined gluteus muscle strengthening and lumbar stabilization exercises plus health education based on clinical judgment regarding suitability for supervised exercise therapy. This naturalistic treatment allocation reflected real-world clinical decision-making processes rather than experimental randomization, providing ecological validity for comparative effectiveness assessment.

Data collection procedure

Comprehensive baseline demographic and clinical data were systematically extracted from existing clinical records and physiotherapy assessment documentation utilizing standardized data abstraction forms developed specifically for this retrospective analysis. Sociodemographic variables abstracted included age, gender, occupational classification, and educational attainment as documented in initial patient intake assessments. Clinical data extraction encompassed detailed pain history, symptom duration, previous treatment exposures, and baseline Oswestry Disability Index (ODI) scores documented at initial physiotherapy consultation [16,17]. The ODI, comprising 10 sections addressing pain intensity and functional limitations in activities of daily living, had been administered as part of routine clinical assessment protocols, with disability severity classified according to standardized scoring criteria as follows: 0-20% indicating mild disability, 21-40% moderate disability, 41-60% severe disability, and 61-80% crippled status.

A retrospective review of treatment records revealed that group A patients had received comprehensive instruction in spine-neutral health education principles encompassing postural awareness training, ergonomic modification strategies, and movement pattern optimization during their clinical physiotherapy sessions. Specific educational components documented in clinical notes included avoidance of slouched sitting postures, maintenance of neutral spinal curves during occupational and recreational activities, correction of poor standing posture, modification of prolonged ambulation patterns, instruction in the golfer's lift technique for load transfer from ground level, application of the archer's bow principle for forward bending activities, and utilization of the saddle toss principle for rotational movements. Clinical documentation indicated that patients received instructions to implement these principles consistently throughout daily activities for the prescribed four-week treatment period.

Group B patients' clinical records documented receipt of identical spine-neutral health education plus supervised instruction in structured exercise protocols performed on alternate days with a frequency of three sessions weekly, as evidenced by physiotherapy attendance logs and treatment documentation. The exercise regimen documented in clinical records comprised core stabilization exercises, including the curl-up maneuver targeting rectus abdominis activation, side bridge positions for lateral trunk stabilization with modifications for patients unable to achieve standard positioning, and the bird dog exercise emphasizing contralateral limb extension while maintaining neutral spinal alignment. Gluteus muscle-strengthening exercises documented included progressive resistance protocols targeting gluteus maximus, medius, and minimus through hip extension, abduction, and external rotation movements, performed under physiotherapist supervision.

Post-treatment ODI assessments were documented in clinical records following completion of the four-week treatment protocol, enabling a retrospective pre-post comparison of functional disability outcomes. Treatment adherence data were extracted from physiotherapy session attendance records and clinical documentation of patient compliance with prescribed protocols.

Data analysis

All retrospectively extracted data were systematically entered into Microsoft Excel (Redmond, WA: Microsoft Corp.) spreadsheets for preliminary organization, quality verification, and validation against source documentation and subsequently transferred to IBM SPSS Statistics software version 26.0 (Armonk, NY: IBM Corp.) for comprehensive statistical analysis. Descriptive statistics were computed for all variables, with continuous data presented as means and standard deviations, and categorical variables expressed as frequencies and percentages. Normality of continuous variable distributions was evaluated using Kolmogorov-Smirnov and Shapiro-Wilk tests. Between-group comparisons of continuous baseline characteristics were performed using independent samples t-tests for normally distributed variables, while categorical variable associations were assessed using chi-square tests with Yates' continuity correction applied for cells with expected frequencies below five. Within-group longitudinal comparisons of pre-intervention and post-intervention ODI scores were analyzed using paired-samples t-tests, with Pearson correlation coefficients computed to evaluate pre-post score relationships. Effect sizes were calculated using Cohen’s D methodology to quantify the magnitude of therapeutic effects independent of sample size considerations. Statistical significance was established at an alpha level of 0.05 for all inferential analyses, with two-tailed probability values reported to four decimal places. Clinical success was operationalized as achievement of a 50% or greater reduction in ODI score from baseline, while complete recovery was defined as a post-intervention ODI score of zero, enabling categorical outcome assessment beyond continuous score analyses.

## Results

The baseline demographic and clinical assessment in Table 1 demonstrated substantial homogeneity between the intervention cohorts, establishing methodological rigor for subsequent comparative analyses. The mean age distribution revealed 28.48 years with a standard deviation of 6.352 years in group A compared to 27.80 years with a standard deviation of 8.114 years in group B, yielding no statistically significant difference (t = 0.345; p = 0.7316). Gender distribution analysis showed that 15 participants (60.00%) in group A were female, compared with 21 participants (84.00%) in group B; chi-square testing revealed a non-significant trend toward female predominance in the combined exercise intervention cohort (χ² = 3.571; p = 0.0590). The pre-intervention ODI scores established comparable baseline functional disability, with group A demonstrating a mean of 12.28 with a standard deviation of 4.971 compared to group B's mean of 13.68 with a standard deviation of 6.511 (t = -0.898; p = 0.3736). These findings confirmed the absence of significant baseline disparities between intervention groups, thereby ensuring the validity of subsequent outcome comparisons and minimizing potential confounding variables in the assessment of therapeutic efficacy.

**Table 1:** Baseline demographic and clinical characteristics of study participants across intervention groups. *Independent samples t-test. **Chi-square test. Data are presented as mean ± standard deviation for continuous variables and n (%) for categorical variables. A p-value of less than 0.05 was statistically significant. SNHE: spine neutral health education training group; SMG + LSE: strengthen muscles of gluteus + lumbar segmental stabilization exercise group; ODI: Oswestry Disability Index

Parameters	Group A (SNHE) (n = 25)	Group B (SMG + LSE) (n = 25)	Test statistic	p-Value
Age (years)	28.48 ± 6.352	27.80 ± 8.114	t = 0.345^*^	0.7316
Gender (female)	15 (60.00%)	21 (84.00%)	χ² = 3.571^**^	0.0590
Gender (male)	10 (40.00%)	4 (16.00%)	χ² = 3.571^**^	0.0590
Pre-intervention ODI score	12.28 ± 4.971	13.68 ± 6.511	t = -0.898^*^	0.3736

The pre-intervention disability severity stratification in Table 2 revealed a predominantly mild-to-moderate functional impairment profile across the study population, with distribution patterns demonstrating statistical equivalence between intervention cohorts. Among participants assigned to group A, 17 individuals (68.00%) exhibited mild disability classification compared to 14 individuals (56.00%) in group B, constituting an aggregate of 31 participants (62.00%) across the total sample with no statistically significant inter-group disparity (χ² = 0.778; p = 0.6777). Moderate disability classification encompassed eight participants (32.00%) in the spine neutral health education cohort compared to 10 participants (40.00%) in the combined exercise intervention group, representing 18 participants (36.00%) of the total study population without demonstrating significant distributional differences (χ² = 0.346; p = 0.5565). Notably, severe disability classification was observed in only a single participant (4.00%) allocated to group B, constituting one individual (2.00%) of the aggregate sample, with statistical analysis precluded by the minimal cell frequency. This baseline disability distribution pattern established that the majority of study participants presented with functional impairments amenable to conservative exercise-based interventions, thereby validating the appropriateness of the selected therapeutic protocols and confirming the representative nature of the study sample for chronic low back pain rehabilitation research.

**Table 2:** Distribution of pre-intervention disability severity classification according to ODI across study groups. *Chi-square test with Yates continuity correction applied for cells with expected frequencies <5. Data are presented as n (%). A p-value of less than 0.05 was statistically significant. SNHE: spine neutral health education training group; SMG + LSE: strengthen muscles of gluteus + lumbar segmental stabilization exercise group; ODI: Oswestry Disability Index

ODI classification	Group A (SNHE) (n = 25)	Group B (SMG + LSE) (n = 25)	Total (n = 50)	χ²	p-Value
Mild disability (0-20%)	17 (68.00%)	14 (56.00%)	31 (62.00%)	0.778^*^	0.6777
Moderate disability (21-40%)	8 (32.00%)	10 (40.00%)	18 (36.00%)	0.346^*^	0.5565
Severe disability (41-60%)	0 (0.00%)	1 (4.00%)	1 (2.00%)	1.020^*^	0.3124

The post-intervention disability severity distribution in Table 3 revealed substantial therapeutic improvements across both intervention modalities, with distinctive patterns suggesting differential efficacy profiles. Complete functional recovery, characterized by no disability classification, was achieved in seven participants (28.00%) receiving spine-neutral health education compared to 12 participants (48.00%) in the combined gluteus strengthening and lumbar stabilization exercise cohort, representing an aggregate of 19 individuals (38.00%) across the total sample, though this clinically meaningful trend did not achieve statistical significance (χ² = 2.186; p = 0.1393). Mild disability persistence was observed in 16 participants (64.00%) from group A compared to 13 participants (52.00%) from group B, constituting 29 individuals (58.00%) of the total study population without demonstrating significant distributional differences (χ² = 0.747; p = 0.3874). Moderate disability classification persisted exclusively in two participants (8.00%) from the health education cohort, with complete resolution observed in the combined exercise intervention group, though statistical analysis revealed non-significant differences (χ² = 2.087; p = 0.1486). Remarkably, severe disability classification was entirely eliminated across both intervention arms, representing complete eradication of high-grade functional impairment. These findings substantiate the therapeutic efficacy of both conservative intervention strategies while suggesting potential superiority of the combined exercise protocol in achieving complete functional restoration.

**Table 3:** Post-intervention disability severity classification and therapeutic response distribution across study groups. *Chi-square test with Yates' continuity correction applied for cells with expected frequencies <5. Data are presented as n (%). A p-value of less than 0.05 was statistically significant. SNHE: spine neutral health education training group; SMG + LSE: strengthen muscles of gluteus + lumbar segmental stabilization exercise group; ODI: Oswestry Disability Index

ODI classification	Group A (SNHE) (n = 25)	Group B (SMG + LSE) (n = 25)	Total (n = 50)	χ²	p-Value
No disability (0%)	7 (28.00%)	12 (48.00%)	19 (38.00%)	2.186^*^	0.1393
Mild disability (1-20%)	16 (64.00%)	13 (52.00%)	29 (58.00%)	0.747^*^	0.3874
Moderate disability (21-40%)	2 (8.00%)	0 (0.00%)	2 (4.00%)	2.087^*^	0.1486
Severe disability (41-60%)	0 (0.00%)	0 (0.00%)	0 (0.00%)	-	-

The intra-group longitudinal analysis in Table 4 revealed substantial and statistically significant reductions in functional disability across both intervention modalities, with the combined exercise protocol demonstrating a superior magnitude of therapeutic effect. Within the spine neutral health education cohort (group A), the mean ODI score decreased from 12.28 with a standard deviation of 4.971 at baseline to 6.60 with a standard deviation of 3.697 post-intervention, yielding a mean reduction of 5.68 points that achieved high statistical significance (t = 8.145; p < 0.0001). Conversely, the combined gluteus strengthening and lumbar stabilization exercise group (group B) demonstrated a pre-intervention mean ODI score of 13.68 with a standard deviation of 6.511 that diminished to 4.76 with a standard deviation of 2.773 following therapeutic intervention, establishing a mean reduction of 8.92 points with profound statistical significance (t = 11.397; p < 0.0001). The magnitude of improvement in group B represented approximately 1.57-fold greater therapeutic efficacy compared to group A, substantiating the synergistic benefits of combined exercise modalities. Additionally, robust positive correlations between pre-intervention and post-intervention ODI scores were observed in both group A (r = 0.827; p < 0.0001) and group B (r = 0.812; p < 0.0001), indicating consistent therapeutic response patterns across baseline disability severity strata and validating the reliability of outcome measurement protocols.

**Table 4:** Comparative analysis of pre-intervention and post-intervention ODI scores within study groups. *Paired samples t-test. **Pearson correlation between pre-intervention and post-intervention ODI scores. Data are presented as mean ± standard deviation. A p-value of less than 0.05 was statistically significant. SNHE: spine neutral health education training group; SMG + LSE: strengthen muscles of gluteus + lumbar segmental stabilization exercise group; ODI: Oswestry Disability Index

Parameters	Group A (SNHE) (n = 25)	p-Value	Group B (SMG + LSE) (n = 25)	p-Value
Pre-intervention ODI (mean ± SD)	12.28 ± 4.971	-	13.68 ± 6.511	-
Post-intervention ODI (mean ± SD)	6.60 ± 3.697	-	4.76 ± 2.773	-
Mean difference (reduction)	5.68	-	8.92	-
Paired t-statistic	8.145	<0.0001^*^	11.397	<0.0001^*^
Pre-post correlation (r)	0.827	<0.0001^**^	0.812	<0.0001^**^

The sociodemographic profiling in Table 5 revealed a predominantly skilled workforce with substantial educational attainment, demonstrating general comparability between intervention cohorts with isolated distributional variations. Occupational stratification established that skilled workers constituted the predominant category, with 19 participants (76.00%) in group A compared to 16 participants (64.00%) in group B, representing an aggregate of 35 individuals (70.00%) across the total sample without statistically significant inter-group disparity (χ² = 0.870; p = 0.3510). Semi-skilled workers comprised four participants (16.00%) in the health education cohort and five participants (20.00%) in the combined exercise intervention group, totaling nine individuals (18.00%) with equivalent distributional patterns (χ² = 0.133; p = 0.7155). The housewife/homemaker classification encompassed one participant (4.00%) from group A compared to four participants (16.00%) from group B, constituting five individuals (10.00%) collectively, though this numerical disparity did not achieve statistical significance (χ² = 2.083; p = 0.1489). Educational attainment analysis revealed that higher secondary education represented the predominant qualification level, with 21 participants (84.00%) in group A and 20 participants (80.00%) in group B, totaling 41 individuals (82.00%) without significant distributional differences (χ² = 0.134; p = 0.7144). Notably, secondary education demonstrated significant distributional disparity, with exclusive representation of four participants (16.00%) in group B compared to complete absence in group A (χ² = 4.348; p = 0.0371), constituting the sole sociodemographic parameter exhibiting statistically significant inter-group variation and potentially warranting consideration in subgroup analyses.

**Table 5:** Sociodemographic distribution of study participants according to occupational classification and educational attainment. *Chi-square test with Yates' continuity correction applied for cells with expected frequencies <5. Data are presented as n (%). A p-value of less than 0.05 was statistically significant. SNHE: spine neutral health education training group; SMG + LSE: strengthen muscles of gluteus + lumbar segmental stabilization exercise group

Parameters	Group A (SNHE) (n = 25)	Group B (SMG + LSE) (n = 25)	Total (n = 50)	χ²	p-Value
Occupational classification					
Housewife/homemaker	1 (4.00%)	4 (16.00%)	5 (10.00%)	2.083^*^	0.1489
Semi-skilled worker	4 (16.00%)	5 (20.00%)	9 (18.00%)	0.133^*^	0.7155
Skilled worker	19 (76.00%)	16 (64.00%)	35 (70.00%)	0.870^*^	0.3510
Unskilled worker	1 (4.00%)	0 (0.00%)	1 (2.00%)	1.020^*^	0.3124
Educational attainment					
Elementary education	2 (8.00%)	1 (4.00%)	3 (6.00%)	0.355^*^	0.5513
Primary education	2 (8.00%)	0 (0.00%)	2 (4.00%)	2.087^*^	0.1486
Secondary education	0 (0.00%)	4 (16.00%)	4 (8.00%)	4.348^*^	0.0371
Higher secondary education	21 (84.00%)	20 (80.00%)	41 (82.00%)	0.134^*^	0.7144

The between-group comparative analysis in Table 6 revealed statistically significant superiority of the combined gluteus strengthening and lumbar stabilization exercise protocol across multiple efficacy parameters, substantiating the synergistic therapeutic benefits of integrated exercise interventions. Post-intervention ODI scores demonstrated a mean of 6.60 with a standard deviation of 3.697 in the spine neutral health education cohort compared to 4.76 with a standard deviation of 2.773 in the combined exercise intervention group, establishing statistically significant between-group disparity favoring the exercise protocol (t = 2.079; p = 0.0433; Cohen's d = 0.588). The percentage reduction from baseline disability scores revealed substantially greater therapeutic efficacy in group B, with a mean reduction of 65.20% with a standard deviation of 14.67% compared to 46.26% with a standard deviation of 18.42% in group A, yielding highly significant differences (t = -4.187; p = 0.0001) and a large effect size (Cohen's d = 1.183). Clinical success rate, operationalized as achievement of ≥50% reduction in ODI score from baseline, was attained by 12 participants (48.00%) in the health education cohort compared to 20 participants (80.00%) in the combined exercise intervention group, demonstrating statistically significant superiority (χ² = 5.791; p = 0.0161) and representing a 1.67-fold greater success rate. Complete functional recovery, defined as a post-intervention ODI score of zero, was achieved by seven participants (28.00%) in group A compared to 12 participants (48.00%) in group B, suggesting a clinically meaningful trend toward superior outcomes despite not achieving statistical significance (χ² = 2.186; p = 0.1393). These comprehensive findings establish robust evidence supporting the integration of gluteus muscle strengthening with lumbar stabilization exercises as a superior therapeutic approach for chronic low back pain management compared to health education alone.

**Table 6:** Between-group comparative analysis of post-intervention ODI scores and therapeutic efficacy assessment. *Independent samples t-test. **Chi-square test. Data are presented as mean ± standard deviation for continuous variables; n (%) for categorical variables. A p-value of less than 0.05 was statistically significant. Clinical success is defined as ≥50% reduction in ODI score from baseline; complete recovery is defined as a post-intervention ODI score of 0. SNHE: spine neutral health education training group; SMG + LSE: strengthen muscles of gluteus + lumbar segmental stabilization exercise group; ODI: Oswestry Disability Index

Parameters	Group A (SNHE) (n = 25)	Group B (SMG + LSE) (n = 25)	Test statistic	p-Value	Effect size (Cohen's d)
Post-intervention ODI score	6.60 ± 3.697	4.76 ± 2.773	t = 2.079^*^	0.0433	0.588
Percentage reduction from baseline	46.26 ± 18.42%	65.20 ± 14.67%	t = -4.187^*^	0.0001	1.183
Clinical success rate (≥50% reduction)	12 (48.00%)	20 (80.00%)	χ² = 5.791^**^	0.0161	-
Complete recovery rate (ODI = 0)	7 (28.00%)	12 (48.00%)	χ² = 2.186^**^	0.1393	-

## Discussion

The present retrospective observational study systematically compared functional disability outcomes between patients receiving combined gluteus muscle strengthening with lumbar stabilization exercises versus spine-neutral health education alone during routine physiotherapy management of chronic low back pain, utilizing the ODI as the primary outcome measure. The comparative analysis demonstrated statistically significant reductions in functional disability across both therapeutic modalities, with the combined exercise protocol exhibiting superior outcomes characterized by a mean ODI score reduction of 8.92 points compared to 5.68 points in the health education cohort (p < 0.0001), representing approximately 1.57-fold greater efficacy. These real-world effectiveness findings substantiate the synergistic benefits of integrating targeted muscular strengthening with segmental stabilization exercises and provide compelling evidence for optimizing conservative management strategies within routine chronic low back pain rehabilitation practice.

The substantial reduction in pain-related disability observed in the combined exercise intervention group aligns consistently with previous investigations examining integrated therapeutic approaches. Jeong et al. conducted a comparable study with 30 participants demonstrating that gluteus muscle strengthening combined with lumbar stabilization exercises produced significant improvements in lumbar muscle strength and balance parameters, corroborating our findings of superior functional outcomes with combined protocols [10]. Similarly, Fukuda et al. reported in their randomized controlled trial of 148 patients that adding hip strengthening exercises to manual therapy and segmental stabilization yielded significantly greater improvements in pain intensity and disability scores compared to segmental stabilization alone, with effect sizes ranging from 0.52 to 0.87, substantiating our observed Cohen's d of 1.183 for percentage disability reduction [14]. The mechanistic rationale underlying these synergistic effects relates to the biomechanical interdependence between lumbopelvic stability and hip muscular function, as extensively documented by Buckthorpe et al., who emphasized that gluteus maximus weakness compromises pelvic stability and perpetuates aberrant movement patterns that exacerbate low back pain symptomatology [11].

Our finding that 80% of participants in the combined exercise group achieved clinical success (≥50% ODI reduction) compared to 48% in the health education cohort demonstrates substantial clinical superiority and aligns with meta-analytic evidence. Gomes-Neto et al. performed a systematic review with meta-analysis encompassing 17 randomized controlled trials with 1,082 participants, demonstrating that stabilization exercises produced significantly greater improvements in pain and disability compared to general exercises or manual therapy, with standardized mean differences of -0.49 for pain and -0.37 for disability, supporting our observed between-group disparities [7]. However, our complete functional recovery rate of 48% in the combined exercise group exceeds the typical remission rates reported in chronic low back pain literature, suggesting particular efficacy of our integrated protocol. Conversely, Bhadauria and Gurudut reported more modest differences in their three-arm trial of 45 participants comparing lumbar stabilization, dynamic strengthening, and Pilates interventions, with all groups demonstrating improvements but without substantial between-group disparities, potentially attributable to differences in intervention intensity, duration, and participant selection criteria [18].

The dose-response relationship observed in our study, wherein alternate-day exercise sessions over four weeks produced clinically significant improvements, warrants consideration in the context of existing evidence on optimal exercise prescription parameters. Mueller and Niederer conducted a systematic review with meta-regression analyzing 22 studies encompassing 1,547 participants to evaluate dose-response relationships of stabilization exercises in chronic non-specific low back pain, demonstrating that exercise frequency of two to three sessions weekly for durations of six to eight weeks produced optimal therapeutic effects with diminishing returns at higher frequencies [8]. Our intervention protocol of three weekly sessions aligns with these recommendations and achieved substantial therapeutic efficacy despite the relatively shorter four-week duration compared to typical eight to 12 week protocols reported in the literature. This finding suggests potential for accelerated therapeutic response with combined exercise modalities, though longer-term follow-up would be necessary to evaluate outcome sustainability. Suh et al. reported in their randomized controlled trial of 40 participants that combining lumbar stabilization exercises with walking interventions over eight weeks produced greater improvements in pain and disability compared to walking alone, with effect sizes comparable to our findings, supporting the principle that multimodal exercise approaches yield superior outcomes through complementary biomechanical and neuromuscular mechanisms [19].

The specific exercise components incorporated in our combined intervention protocol, curl-up, side bridge, and bird dog exercises, represent evidence-based core stabilization techniques with established efficacy profiles. Cho et al. investigated lumbar stabilization exercises in 30 chronic low back pain patients over four weeks, demonstrating significant improvements in functional disability and lumbar lordosis angle with mean ODI reductions of 7.2 points, closely approximating our observed effects [5]. The bird dog exercise, which featured prominently in our protocol, has been specifically validated for enhancing neuromuscular control of the lumbar spine while minimizing compressive forces. Moon et al. compared lumbar stabilization exercises to dynamic lumbar strengthening in 50 patients with chronic low back pain, reporting that stabilization exercises produced superior improvements in pain intensity and functional disability with mean ODI reductions of 8.4 points in the stabilization group compared to 5.1 points in the strengthening group, paralleling our finding of superior efficacy with stabilization-focused protocols [20]. The integration of gluteus strengthening with these core stabilization exercises in our protocol appears to have provided synergistic benefits through simultaneous enhancement of both local segmental stabilizers and global movement system musculature.

The substantial therapeutic efficacy observed in our spine-neutral health education group, with a mean ODI reduction of 5.68 points, merits particular consideration, as it exceeds the minimal clinically important difference threshold and contradicts prevailing assumptions about passive educational interventions. These findings parallel recent evidence emphasizing the importance of self-management strategies in chronic low back pain rehabilitation. Bourke et al. conducted qualitative research with 18 participants exploring patient experiences of self-management, revealing that education regarding posture, movement patterns, and pain neuroscience significantly influenced therapeutic outcomes through enhanced self-efficacy and behavioral modification [21]. Similarly, Zhou et al. performed a narrative review synthesizing evidence on self-management interventions, concluding that structured educational programs incorporating movement retraining and postural awareness constitute essential components of comprehensive chronic low back pain management, validating our inclusion of spine-neutral health principles as an active therapeutic intervention rather than a passive control condition [22].

The principles of spine neutral health education implemented in our study, including the golfer's lift technique, archer's bow principle, and saddle toss principle, represent evidence-based movement strategies designed to minimize aberrant loading patterns and optimize biomechanical efficiency during functional activities. Heo et al. investigated the combined effects of lumbar stabilization exercises and thoracic mobilization in 32 patients with chronic low back pain, demonstrating that addressing movement patterns across multiple spinal segments produced greater therapeutic benefits than isolated lumbar interventions, with mean pain reductions of 3.4 points on the visual analog scale [23]. This finding supports our comprehensive approach, integrating both exercise interventions and movement education. Additionally, Kang et al. evaluated lumbar stabilization exercises performed on stable versus unstable surfaces in 40 automobile assembly workers with chronic low back pain over six weeks, reporting significant improvements in pain and disability across both conditions with mean ODI reductions of 6.8 points, suggesting that the core principles of stabilization training possess therapeutic efficacy independent of specific equipment or surface conditions, thereby enhancing practical applicability in diverse clinical and home-based settings [24].

The gender distribution in our study revealed predominant female representation, particularly in the combined exercise intervention group (84%), reflecting established epidemiological patterns in chronic low back pain prevalence. Wu et al. analyzed Global Burden of Disease data demonstrating that low back pain prevalence and disability rates exhibit female predominance across most age groups and geographic regions, with age-standardized prevalence rates approximately 1.2-fold higher in females compared to males [1]. This gender disparity potentially relates to biomechanical factors, including pelvic morphology, hormonal influences on connective tissue properties, and occupational exposure patterns, as discussed by Ferreira et al. in their comprehensive analysis projecting that low back pain burden will continue escalating through 2050 with persistent gender-based disparities [2]. Our finding of non-significant gender distributional differences between groups (p = 0.0590) suggests adequate randomization despite the trend toward female predominance, though future investigations should consider gender-stratified analyses to elucidate potential sex-specific therapeutic response patterns.

The hip muscle strengthening component of our intervention protocol addresses an increasingly recognized biomechanical contributor to chronic low back pain pathophysiology. Santamaría et al. conducted a systematic review of nine studies encompassing 526 participants examining hip muscle strengthening effects on non-specific low back pain, demonstrating that hip strengthening interventions produced significant reductions in pain intensity and disability with pooled effect sizes ranging from 0.45 to 0.78, supporting the integration of hip-focused exercises into comprehensive low back pain rehabilitation protocols [12]. The gluteus medius, in particular, plays a critical role in frontal plane pelvic stability during single-limb support phases of gait and functional activities. Ahn et al. investigated gluteal muscle strengthening exercise-based core stabilization training in 60 patients with chronic low back pain over eight weeks, reporting significant improvements in pain intensity, functional disability, and quality of life measures with mean ODI reductions of 9.2 points, closely approximating our observed therapeutic effects and further validating the efficacy of combined gluteal and core stabilization approaches [13].

The complete elimination of severe disability classification across both intervention groups represents a clinically significant finding that underscores the efficacy of conservative therapeutic approaches in high-grade functional impairment. This outcome aligns with clinical practice guideline recommendations emphasizing exercise-based interventions as first-line management strategies. Nicol et al. synthesized recent international guidelines for chronic low back pain management, identifying consistent recommendations across North American, European, and Asian clinical practice standards prioritizing non-pharmacological interventions, including structured exercise programs, patient education, and multimodal rehabilitation approaches [4]. Similarly, Zhou et al. performed a global comparison of clinical practice guidelines demonstrating convergent recommendations for exercise therapy as a cornerstone intervention, with specific endorsement of motor control exercises and stabilization protocols for patients with movement control impairments, directly supporting our therapeutic approach [3].

The robust positive correlations between pre-intervention and post-intervention ODI scores (r = 0.827 in group A; r = 0.812 in group B) indicate consistent therapeutic response patterns across baseline disability severity strata, suggesting the broad applicability of our intervention protocols. This finding contrasts somewhat with research by Larivière et al., who analyzed 132 patients participating in lumbar stabilization programs and identified distinct responder subgroups characterized by differential improvement trajectories, with larger improvement subgroups demonstrating greater reductions in pain catastrophizing and kinesiophobia [25]. The consistency of therapeutic response in our study may reflect our specific inclusion criteria targeting mild-to-moderate disability (ODI ≥5) and our exclusion of severe disability cases, potentially limiting generalizability to more complex chronic low back pain presentations.

The occupational distribution in our study revealed a predominance of skilled workers (70%), potentially influencing therapeutic adherence and outcome trajectories. Rainville et al. investigated exercise as treatment for chronic low back pain in 89 participants, demonstrating that occupational demands and physical work requirements significantly moderated therapeutic outcomes, with sedentary workers exhibiting greater functional improvements compared to heavy laborers [26]. Our finding of substantial therapeutic efficacy across occupational categories suggests that both spine-neutral health education and combined exercise interventions possess applicability across diverse vocational contexts, though future research should examine occupation-specific modification protocols to optimize therapeutic delivery.

Clinical significance

The clinical significance of these retrospective comparative findings extends beyond statistical parameters to encompass substantial implications for chronic low back pain management protocols and healthcare resource allocation strategies. The observed clinical success rate of 80% among patients receiving combined gluteus strengthening with lumbar stabilization exercises, compared to 48% receiving health education alone, represents a number needed to treat of approximately 3.1, indicating that for every three patients managed with the combined exercise protocol rather than education alone, one additional patient achieves clinically meaningful improvement. This therapeutic efficiency, documented in routine clinical practice, possesses particular relevance for resource-constrained healthcare settings where optimization of real-world intervention effectiveness directly influences patient throughput and cost-effectiveness ratios. Furthermore, the complete functional recovery rate of 48% observed in the combined exercise cohort substantially exceeds typical remission rates reported in epidemiological studies of chronic low back pain, suggesting potential for long-term disability reduction and secondary prevention of recurrent episodes. The therapeutic efficacy documented across both management approaches, with complete elimination of severe disability classifications, substantiates the appropriateness of conservative physiotherapy as first-line treatment for chronic low back pain, potentially reducing reliance on pharmacological interventions and invasive procedures. These retrospective effectiveness data provide compelling evidence supporting the integration of structured exercise programs combining gluteus muscle strengthening with lumbar stabilization exercises into routine clinical physiotherapy protocols.

Strengths of the study

This retrospective observational study encompasses several substantive strengths, enhancing the validity and reliability of findings. The use of validated outcome measurement instruments, specifically the ODI, which has established psychometric properties and widespread clinical acceptance, ensures standardized disability assessment in routine practice and facilitates comparability with the existing literature. The analysis of structured therapeutic protocols with clearly defined exercise parameters and health education components documented in clinical records enhances reproducibility and practical applicability. The comprehensive demographic characterization, including occupational classification and educational attainment, enables contextual interpretation of real-world therapeutic outcomes and identification of potential moderating variables. The statistical analytical approach, incorporating parametric and non-parametric techniques with appropriate corrections, demonstrates analytical rigor. The adequate sample size based on power calculations and balanced group allocation ensures statistical power for comparative effectiveness assessments while providing ecological validity through naturalistic treatment assignment reflecting actual clinical decision-making processes.

Limitations

Several methodological constraints inherent to retrospective observational designs warrant acknowledgment when interpreting findings and considering generalizability. The absence of randomized treatment allocation introduces potential selection bias and unmeasured confounding variables, as therapeutic assignment reflected clinical decision-making rather than probability-based random assignment, despite achievement of baseline demographic equivalence. The retrospective nature precludes prospective standardization of data collection procedures, outcome assessment timing, and intervention fidelity monitoring, relying upon clinical documentation quality generated during routine care delivery. The relatively short treatment observation period of one month, while demonstrating significant therapeutic efficacy, precludes evaluation of long-term outcome sustainability and relapse rates. The single-center data derivation limits external validity and generalizability to other clinical settings with different patient populations or physiotherapy protocols. The six-month observation period, while adequate for sample size achievement, represents a relatively compressed temporal window that may not capture seasonal variations in patient presentation patterns, treatment adherence, or clinical outcomes across extended timeframes. The exclusive reliance on self-reported outcome measures abstracted from clinical records, without incorporation of objective functional assessments, biomechanical measurements, or muscle activation parameters, limits comprehensive evaluation of therapeutic mechanisms underlying observed functional improvements and precludes assessment of intervention fidelity.

Recommendations

Future prospective randomized controlled trials should implement adequate allocation concealment and assessor blinding to minimize bias and enhance internal validity beyond retrospective comparative analyses. Longitudinal follow-up assessments extending six to 12 months post-treatment would elucidate outcome sustainability and identify optimal maintenance protocols. Integration of objective outcome measures, including surface electromyography for muscle activation assessment, three-dimensional motion analysis for movement pattern evaluation, and magnetic resonance imaging for muscle morphology quantification, would provide mechanistic insights. Investigation of dose-response relationships examining treatment frequency, duration, and intensity parameters would optimize exercise prescription protocols. Prospective multi-center observational studies capturing real-world effectiveness data across diverse clinical settings would enhance external validity and generalizability of findings.

## Conclusions

This retrospective observational study provides strong real-world evidence that chronic low back pain patients who do both gluteus muscle strengthening and lumbar stabilization exercises during routine physiotherapy management have better functional disability outcomes than those who only do spine-neutral health education. The combined exercise protocol achieved statistically significant reductions in functional disability with large effect sizes, higher clinical success rates, and complete functional recovery in nearly half of the participants, substantially exceeding outcomes observed with health education alone. Both therapeutic modalities demonstrated significant improvements, complete elimination of severe disability classifications, and favorable tolerability profiles documented within routine clinical practice, substantiating the appropriateness of conservative physiotherapy as first-line treatment. The synergistic benefits observed with combined targeted muscular strengthening and segmental stabilization exercises appear to derive from enhanced lumbopelvic stability, improved neuromuscular control, and optimized movement patterns through biomechanically complementary mechanisms. These retrospective comparative effectiveness findings possess substantial clinical significance for rehabilitation practice and healthcare resource allocation, supporting the integration of structured combined exercise programs into evidence-based clinical physiotherapy protocols while highlighting the continued importance of patient education and self-management strategies in comprehensive multimodal chronic low back pain rehabilitation.
